# Trends in Prevalence of Dyslipidaemias and the Risk of Mortality in Lithuanian Urban Population Aged 45–64 in Relation to the Presence of the Dyslipidaemias and the Other Cardiovascular Risk Factors

**DOI:** 10.1371/journal.pone.0100158

**Published:** 2014-06-23

**Authors:** Dalia Luksiene, Abdonas Tamosiunas, Migle Baceviciene, Ricardas Radisauskas, Vilija Malinauskiene, Anne Peasey, Martin Bobak

**Affiliations:** 1 Institute of Cardiology, Academy of Medicine, Lithuanian University of Health Sciences, Kaunas, Lithuania; 2 Department of Epidemiology and Public Health, University College London, London, United Kingdom; Universität Bochum, Germany

## Abstract

The aim of this study was to provide reliable information on dyslipidaemias, to estimate the trend of the prevalence of dyslipidaemias and other selected cardiovascular disease (CVD) risk factors at population level, and to evaluate the risk of all-cause and CVD mortality in relation to presence of mixed dyslipidaemias and other CVD risk factors.

**Methods:**

Data from the five surveys (1983–2008) are presented. A random sample of 9,209 subjects aged 45–64 was selected for statistical analysis. During follow-up there were 1653 death cases from any cause, 864 deaths from CVD. Estimates of hazard ratios (HR) and 95% confidence intervals (CI) were based on the multivariate Cox proportional hazards regression for all-cause mortality and CVD mortality.

**Results:**

During 25 year period the prevalence of normal total cholesterol level (<5.2 mmol/L) significantly increased only in women; triglycerides and high density lipoprotein (HDL) cholesterol did not change in men and women. Findings in our longitudinal study showed that in men and women mixed dyslipidaemias (HDL cholesterol <1.03 mmol/L plus triglycerides ≥1.70 mmol/L) significantly increased the risk for all-cause and CVD mortality (respectively in men HR = 1.30; HR = 1.15, in women HR = 1.83; HR = 2.13). These mixed dyslipidaemia combinations combination with the other risk factors such as arterial hypertension, high fasting glucose level increased all-cause and CVD mortality risk in men and women; while, these mixed dyslipidaemias plus smoking increased all-cause and CVD mortality risk only in men compared to never smokers without these dyslipidaemias (respectively HR = 1.89; HR = 1.92); and these dyslipidaemias plus obesity increased all-cause and CVD mortality risk in women (respectively HR = 2.25; HR = 2.39) and CVD mortality risk in men (HR = 1.72), as compared to responders without obesity and these dyslipidaemias.

**Conclusion:**

Mixed dyslipidaemias (reduced HDL cholesterol plus elevated triglycerides) significantly increased the risk for all-cause and CVD mortality in this Lithuanian population aged 45–64 years.

## Introduction

Cardiovascular disease (CVD) is now the leading cause of death worldwide; it is on the rise and has become a true pandemic that respects no borders [1]. In Lithuania, CVD incidence and mortality rates both among men and women are higher than in most European countries, especially when compared to high-income western European countries [2], [3]. In 2010, the age standardized mortality from coronary heart disease (CHD) for Lithuanian men aged 25–64 years was 198.2 per 100 000 population and for women 44.0 per 100 000 population, while the average rate in the European Union was 102.0 per 100 000 population for men and 24.8 per 100 000 population for women of comparable age [4]. Dyslipidaemias represent one of the major of known CVD risk factors [1]. Hence, European guidelines for reducing CVD risk and current clinical practice guidelines main focus on lowering serum levels of total cholesterol and low density lipoprotein (LDL) cholesterol [5]. However, other lipids abnormalities such as low serum levels of high-density lipoprotein (HDL) cholesterol and elevated levels of triglycerides also increase the risk of CVD [6]–[9]. Serum levels of HDL cholesterol and triglycerides tend to be inversely correlated, likely reflecting their respective participation in lipid transport and reverse cholesterol transport [10]. This suggests that the separate dyslipidaemias of low HDL cholesterol and elevated triglycerides may be viewed in combination [6], [11]. Despite national lipid guidelines, the prevalence of these abnormal lipid parameters alone or in combination (mixed dyslipidaemia) is not well recognized [12].

Some main risk factors of CVD such as smoking, arterial hypertension (AH), hyperglycaemia, obesity, are associated with lipids level in blood serum [13]–[15]. The data from 27 general populations of the WHO MONICA Project confirms the role of smoking in women, as independent risk factors for dyslipidaemia [13]. Data from the population-based Munster Heart Study (PROCAM) show that smoking was more strongly associated with low HDL cholesterol than with high total cholesterol level [14]. Obesity increases cardiovascular risk through risk factors such as increased fasting plasma triglycerides, high LDL cholesterol, low HDL cholesterol, elevated blood glucose and insulin levels and high blood pressure (BP) [15]. The distinctive dyslipidaemia profile of obesity sees increased triglycerides, decreased HDL cholesterol and normal or slightly increased LDL cholesterol [15]. The data from nine diabetic population samples from the WHO Multinational Study show role of circulating glucose and triglycerides concentration and their interactions with other CVD risk factors [16]. However, there is a lack of the data regarding the role of combined effect of mixed dyslipidaemias and other main CVD risk factors on all-cause and CVD mortality risk in Lithuania.

In this investigation, we have two primary goals: (1) to provide reliable information on dyslipidaemias, such as, estimate the trend of the prevalence of dyslipidaemias and other selected CVD risk factors in population level; and (2) to evaluate the risk of all-cause and CVD mortality in relation to the presence of the mixed dyslipidaemias and other CVD risk factors among 45–64 years urban population from 1983 to 2011.

## Materials and Methods

### Ethics Statement

All five studies were approved by the Regional Biomedical Research Ethics Committee at the Lithuanian University of Health Sciences and the HAPIEE study (2006–2008) - also by the UCLH Research Ethics Committee Alpha at University College London, UK. All respondents provided written informed consent.

### Study sample

Data from the five surveys are presented in the article. The first three surveys in the framework of the Multinational Monitoring of Trends and Determinants in Cardiovascular Disease (MONICA) study were performed in 1983–1984, 1986–1987 and 1992–1993, respectively. The fourth survey was conducted in 2001–2002 in accordance with MONICA protocol. The fifth survey was performed in 2006–2008 in the framework of the Health, Alcohol and Psychosocial Factors in Eastern Europe (HAPIEE) study [17]. These surveys were carried out in Kaunas city with the population size of 348,624. All five random samples of men and women aged 45–64, stratified by gender and age, were randomly selected from the Kaunas population register data and screened in 10-year age groups (45–54 and 55–64 years of age). The response rates were for the first survey −70.2%, for the second −69.6%, for the third −58.6%, for the fourth −62.4%, and for the fifth −58.1%. Finally, a sample of 9,209 subjects was assigned for statistical analysis.

### Baseline health examination

In each survey, the measurements of BP, weight, height, and laboratory analyses were conducted using the same methodology. The information and variables determined using the questionnaire was based on same or comparable questionnaires.

### Measurements

BP was measured two times using mercury sphygmomanometer and appropriately sized arm cuffs on the right arm. The initial measurement was performed after five minutes of rest. After two minutes, the second measurement was made. The Korotkoff phase 1 (beginning of the sound) and the fifth phase of Korotkoff (disappearance of the sound) was recorded as systolic and diastolic BP. The mean of the two readings was used. AH was defined as mean systolic BP of at least 140 mmHg or mean diastolic BP of at least 90 mmHg, or both, and/or affirming that respondent has been taking medicine for high BP in the last two weeks. Weight and height were measured with a calibrated medical scale, and without shoes or heavy clothes. Body mass index (BMI) was calculated as the weight in kilograms divided by the height in meters squared (kg/m^2^). Normal weight was defined as BMI <25.0 kg/m^2^, overweight as BMI 25.0–29.99 kg/m^2^ and obesity as BMI ≥30.0 kg/m^2^.

### Laboratory analyses

Biochemical analyses were done for participants on an empty stomach. Serum samples from the first four surveys were analysed in the Laboratory of the Institute of Cardiology of the Lithuanian University of Health Sciences and serum samples from the fifth survey - centrally in one batch in the WHO Regional Lipid Reference Centre, Institute of Clinical and Experimental Medicine, Prague (Czech Republic). Lipid concentrations in serum were measured, using a conventional enzymatic method. Dyslipidaemia were defined by using the National Cholesterol Educational Program (NCEP-ATPIII) criteria [18]. The subjects were classified into three groups according to their total serum cholesterol level: normal (<5.2 mmol/L), increased (5.2–6.19 mmol/L), and high (≥6.2 mmol/L). Reduced HDL cholesterol level was defined as HDL cholesterol <1.03 mmol/L; elevated triglycerides level was defined as ≥1.70 mmol/L. Concentration of glucose in capillary blood was determined by an individual glucometer “Glucotrend” [19]. High level was defined as fasting glucose level ≥6.10 mmol/L.

### Variables determined using the questionnaire

The standard questionnaire included questions regarding the respondent's age, education, smoking status, physical activity, etc. Education was classified into four education levels: primary, incomplete secondary, secondary or college, and university.

Smoking habits were assessed according to the current smoking status. The respondents were classified to three groups: current smokers, former smokers and never smokers. A subject who smoked at least one cigarette per day was classified as current smoker. Physical activity was assessed using a short questionnaire, asking about physically demanding activities in a typical week on summer and winter seasons: active transportation to work and physically demanding activities, such as housework, gardening, and maintenance of the house also engagement in sports, games or hiking. Subjects who were physically active less than 10 hours a week were classified as physically inactive.

### Follow-up

The participants were followed-up from the beginning of each baseline health examination until December 31, 2011 and mortality data were extracted from the regional mortality register. The two outcome measures of interest were all-cause mortality and CVD mortality (without documented history of CVD at entry). All-cause mortality was defined as: 001-E999 – codes of 9th revision of International Classification of Diseases (ICD) (until January 1st 1997); and A00-Z99 – codes of 10th ICD (after January 1st 1997). CVD mortality included CHD, stroke, and other vascular causes and was defined as (390–458 – codes of 9th ICD and I00–I99 – codes of 10th ICD). There were a total of 1653 deaths from any cause (999 men and 654 women), 864 deaths from CVD (521 men and 343 women), and 594 deaths from CVD (without previous CVD at entry) (357 men and 237 women). The mean duration and standard deviation of follow-up was 13.0±9.3 years among men and 13.5±9.5 years among women.

### Statistical analysis

All the analyses were performed separately for men and women. Descriptive statistics (prevalence rates, means and standard deviations (SD)) were calculated for variables in each survey. All surveys were age-adjusted to Kaunas population census of 2006. In 2006, 55% of adults were aged from 45 to 54 years and 45% were 55–64 years of age, with weights ranging between 0.55 and 0.45, which were used in calculating the prevalence. Weights were calculated by dividing the coefficient for each age group by the sum of coefficients for both age groups in each survey. Weighted linear regression to assess time trends from 1983 to 2008 was performed using mean values or percentages, and p<0.05 was defined as statistically significant.

The estimates of hazard ratio (HR) and 95% confidence intervals (CI) were based on the multivariate Cox proportional hazards regression for all-cause mortality and for CVD mortality (without CVD at the entry). Two multivariate Cox proportional hazards regression models in linked mortality file (1983–2008) are presented. The first model (Model 1) included age, study survey year and lipids levels (total cholesterol, triglycerides and HDL cholesterol). The second model (Model 2) included age, study survey year, education, BMI, BP level, fasting glucose level, smoking and physical activity habits and lipids levels (total cholesterol, triglycerides and HDL cholesterol).

The risk of all-cause mortality and mortality from CVD (without CVD at the entry) in an urban population aged 45–64 years in relation to the presence of the dyslipidaemias and a combination of other cardiovascular risk factors (AH, high fasting glucose level, current or former smoking and obesity) was investigated using Cox proportional hazards regression analyses. HRs were adjusted by age, study survey year, education, BMI, total cholesterol level, triglycerides, HDL cholesterol level, BP levels, fasting glucose level, smoking and physical activity habits. All analyses were carried out using SPSS version 13.

## Results

Baseline characteristics of five cohorts aged 45–64 years are presented in separately for men (Table 1) and women (Table 2). All the cohorts were homogenous according to age and gender structure. During the period 1983–2008 the level of education in Kaunas population significantly increased. In men during 25 year period the prevalence of overweight decreased, while the prevalence of obese men increased. Meanwhile generally negative changes in cardiovascular risk profile were observed in men and in women groups (Table 1 and Table 2). In men the mean fasting glucose level increased from 4.72 (1.21) to 5.77 (1.21) mmol/L and in women increased from 4.50 (1.16) to 5.76 (1.08) mmol/L comparing 1983–1984 and 2006–2008 samples (p<0.05). Similarly, the prevalence of high fasting glucose level (≥6.1 mmol/L) significantly increased in both men and women. In women over this 25-year period the prevalence of current smokers increased from 3.4 to 15.4% (p = 0.052). Overall, during 25 year period, risk profile of AH, smoking habits, physical activity habits, serum total cholesterol, triglycerides and HDL cholesterol did not change in men group. The risk profile of AH, physical activity habits, serum triglycerides and HDL cholesterol did not change in women group during this period.

**Table 1:** Characteristics* of men aged 45–64 years in 5 cohorts.

	1983–1984	1986–1987	1992–1993	2001–2002	2006–2008	P for trend
Characteristics* of men	(N = 777)	(N = 603)	(N = 416)	(N = 432)	(N = 2001)	
**Mean age, years** (SD)	53.8 (5.36)	52.9 (5.26)	53.4 (5.86)	53.4 (5.55)	55.0 (5.63)	0.281
**Arterial hypertension (AH)**, %,(95%CI)	58.0 (54.5–61.5)	52.1 (48.1–56.1)	57.8 (53.1–62.5)	58.7 (54.1–63.3)	71.0 (69.0–73.0)	0.134
**BMI, kg/m** ^2^, %, (95%CI)						
<25.0	23.3 (20.3–26.3)	22.4 (19.1–25.7)	30.6 (26.2–35.0)	27.6 (23.4–31.8)	23.2 (21.4–25.0)	0.736
25.0–29.9	50.7 (47.2–54.2)	50.1 (46.1–54.1)	49.9 (45.1–54.7)	44.5 (39.8–49.2)	43.6 (41.4–45.8)	**0.009**
≥30.0	26.0 (22.9–29.1)	27.5 (23.9–31.1)	19.5 (15.7–23.3)	27.8 (23.6–32.0)	33.2 (31.1–35.3)	0.377
**Smoking status,** % (95%CI)						
Current	33.8 (30.5–37.1)	33.9 (30.1–37.7)	28.6 (24.3–32.9)	39.4 (34.8–44.0)	38.9 (36.7–40.9)	0.241
Former	28.4 (25.2–31.6)	21.1 (17.8–24.4)	25.5 (21.3–29.7)	25.1 (21.0–29.2)	28.1 (26.1–30.1)	0.666
Never	37.8 (34.4–41.2)	45.0 (41.0–49.0)	45.9 (41.1–50.7)	35.5 (31.0–40.0)	33.0 (30.9–35.1)	0.251
**Physical activity,** %(95%CI)						
Physically inactive	56.8 (53.3–60.3)	62.2 (58.3–66.1)	37.5 (32.8–42.2)	34.3 (29.8–38.8)	35.3 (33.2–37.4)	0.057
**Level of education,** % (95%CI)						
Primary	20.3 (17.5–23.1)	14.1 (11.3–16.9)	10.3 (7.4–13.2)	1.2 (0.2–2.2)	2.0 (1.4–2.6)	**0.006**
Unfinished secondary	25.9 (22.8–29.0)	28.8 (25.2–32.4)	24.0 (19.9–28.1)	10.7 (7.8–13.6)	27.6 (25.6–29.6)	0.489
Secondary or college	30.7 (27.5–33.9)	30.6 (26.9–34.3)	38.5 (33.8–43.2)	59.8 (55.2–64.4)	36.7 (34.6–38.8)	0.267
University	23.1 (20.1–26.1)	26.5 (23.0–30.0)	27.2 (22.9–31.5)	28.4 (24.1–32.7)	33.6 (31.5–35.7)	**0.032**
**Total cholesterol (TC)**						
Mean (SD), mmol/L	6.00 (1.33)	6.05 (1.07)	5.95 (1.17)	6.19 (1.26)	5.84 (1.10)	0.790
<5.2 mmol/L, % (95%CI)	23.9 (20.4–27.4)	21.4 (18.1–24.7)	27.3 (23.0–31.6)	22.4 (18.5–26.3)	29.1 (27.1–31.1)	0.409
5.2–6.19 mmol/L, % (95%CI)	35.8 (31.9–39.7)	35.9 (32.1–39.7)	36.0 (31.3–40.7)	33.3 (28.8–37.8)	37.1 (35.0–39.2)	0.931
≥6.2 mmol/L, % (95%CI)	40.4 (36.4–44.4)	42.8 (38.7–46.9)	36.7 (32.0–41.4)	44.4(39.7–49.1)	33.9 (31.8–36.0)	0.562
**Triglycerides (TG)**						
Mean (SD), mmol/L	1.68 (1.34)	1.44 (0.84)	1.46 (0.93)	1.63 (0.93)	1.53 (1.10)	0.951
Elevated TG level, % (95%CI)	28.1 (24.4–31.8)	24.5 (20.9–28.1)	28.6 (24.2–33.0)	36.2 (31.6–40.8)	29.1 (27.1–31.1)	0.184
**HDL cholesterol**						
Mean (SD), mmol/L	1.35 (0.37)	1.26 (0.44)	1.19 (0.37)	1.43 (0.39)	1.41 (0.38)	0.314
Reduced HDL chol level, % (95%CI)	19.4 (16.0–22.8)	34.9 (30.9–38.9)	34.7 (30.0–39.4)	12.3 (9.1–15.5)	13.6 (12.1–15.1)	0.272
**Fasting glucose (FG)**						
Mean (SD), mmol/L	4.72 (1.21)	5.13 (1.07)	5.34 (1.53)	5.57 (1.06)	5.77 (1.21)	**0.010**
High FG level, % (95%CI)	7.3 (4.7–9.9)	12.8 (10.1–15.5)	26.0 (21.7–30.3)	14.1 (10.8–17.4)	26.5 (24.5–28.5)	**0.082**

*age-standardized.

Abbreviations: CI – confidence interval; HDL – high density lipoprotein; BMI – body mass index, SBP – systolic blood pressure; DBP – diastolic blood pressure; SD – standard deviation. Reduced HDL cholesterol level (<1.03 mmol/L); Elevated TG level (≥1.70 mmol/L); High FG level (≥6.10 mmol/L); AH (SBP ≥140 and/or DBP ≥90 mm Hg and/or take medication for high blood pressure for at least 2 weeks).

**Table 2:** Characteristics* of women aged 45–64 years in 5 cohorts.

	1983–1984	1986–1987	1992–1993	2001–2002	2006–2008	P for trend
Characteristics* of women	(N = 905)	(N = 589)	(N = 432)	(N = 569)	(N = 2485)	
**Mean age, years (SD)**	53.5 (5.36)	52.8 (5.77)	53.3 (5.43)	52.9 (5.45)	55.0 (5.63)	0.341
**Arterial hypertension (AH),** % (95%CI)	61.0 (57.8–64.2)	50.4 (46.4–54.4)	55.8 (51.1–60.5)	51.0 (46.9–55.1)	54.6 (52.6–56.6)	0.505
**BMI, kg/m^2^,** %(95%CI)						
<25.0	12.8 (10.6–15.0)	15.6 (12.7–18.5)	26.5 (22.3–30.7)	22.0 (18.6–25.4)	24.1 (22.4–25.8)	0.156
25.0–29.9	33.4 (30.3–36.5)	35.0 (31.1–38.9)	34.4 (29.9–38.9)	32.0 (28.2–35.8)	36.0 (34.1–37.9)	0.862
≥30.0	53.7 (50.4–57.0)	49.4 (45.4–53.4)	39.1 (34.5–43.7)	46.0 (41.9–50.1)	39.8 (37.9–41.7)	0.180
**Smoking status,** %(95%CI)						
Current	3.4 (2.2–4.6)	2.9 (1.5–4.3)	2.3 (0.9–3.7)	8.6 (6.3–10.9)	15.4 (14.0–16.8)	0.052
Former	2.1 (1.2–3.0)	1.2 (0.3–2.1)	3.0 (1.4–4.6)	6.0 (4.0–8.0)	9.2 (8.1–10.3)	**0.015**
Never	94.5 (93.0–96.0)	95.9 (94.3–97.5)	94.7 (92.6–96.8)	85.4 (82.5–88.3)	75.4 (73.7–77.1)	**0.033**
**Physical activity,**%(95%CI)						
Physically inactive	50.4 (47.1–53.7)	54.6 (50.6–58.6)	33.9 (29.4–38.4)	39.2 (35.2–43.2)	19.1 (17.6–20.6)	0.064
**Level of education,** %(95%CI)						
Primary	28.5 (25.6–31.4)	18.9 (15.7–22.1)	9.5 (6.7–12.3)	1.6 (0.6–2.6)	1.5 (1.0–2.0)	**0.012**
Unfinished secondary	26.4 (23.5–29.3)	25.5 (22.0–29.0)	19.2 (15.5–22.9)	6.9 (4.8–9.0)	34.8 (32.9–36.7)	0.890
Secondary or college	30.5 (27.5–33.5)	34.7 (30.9–38.5)	44.2 (39.5–48.9)	60.6 (56.6–64.6)	27.7 (25.9–29.5)	0.636
University	14.6 (12.3–16.9)	20.9 (17.6–24.2)	27.1 (22.9–31.3)	30.9 (27.1–34.7)	36.0 (34.1–37.9)	**0.006**
**Total cholesterol (TC)**						
Mean (SD), mmol/L	6.25 (1.20)	6.53 (1.23)	6.51 (1.42)	6.41 (1.38)	6.08 (1.14)	0.453
<5.2 mmol/L, % (95%CI)	13.9 (11.2–16.6)	12.8 (10.1–15.5)	17.6 (14.0–21.2)	18.5 (15.3–21.7)	22.1 (20.5–23.7)	**0.017**
5.2–6.19 mmol/L, % (95%CI)	39.2 (35.4–43.0)	28.2 (24.5–31.9)	27.1 (22.9–31.3)	31.1 (27.3–34.9)	35.6 (33.7–37.5)	0.967
≥6.2 mmol/L, % (95%CI)	46.9 (43.0–50.8)	58.9 (54.9–62.9)	55.3 (50.6–60.0)	50.4 (46.3–54.5)	42.3 (40.4–44.2)	0.373
**Triglycerides (TG)**						
Mean (SD), mmol/L	1.35 (0.75)	1.41 (0.65)	1.25 (0.67)	1.51 (0.91)	1.40 (0.89)	0.485
Elevated TG level, %(95%CI)	19.4 (16.3–22.5)	23.5 (20.0–27.0)	18.1 (14.4–21.8)	29.9 (26.1–33.7)	23.8 (22.1–25.5)	0.170
**HDL cholesterol**						
Mean (SD), mmol/L	1.51 (0.37)	1.49 (0.44)	1.35 (0.36)	1.57 (0.38)	1.60 (0.37)	0.379
Reduced HDL chol level, %(95%CI)	7.8 (5.5–10.1)	14.0 (11.1–16.9)	15.8(12.2–19.4)	6.5 (4.4–8.6)	5.0 (4.1–5.9)	0.337
**Fasting glucose (FG)**						
Mean (SD), mmol/L	4.50 (1.16)	5.01 (0.84)	5.38 (1.33)	5.62 (1.71)	5.76 (1.08)	**0.016**
High FG level, %(95%CI)	5.6 (3.4–7.8)	7.3 (5.2–9.4)	16.7 (13.2–20.2)	11.6 (9.0–14.2)	25.4 (23.7–27.1)	**0.018**

*age-standardized.

Abbreviations: CI – confidence interval; HDL – high density lipoprotein; BMI – body mass index, SBP – systolic blood pressure; DBP – diastolic blood pressure; SD – standard deviation. Reduced HDL cholesterol level (<1.03 mmol/L); Elevated TG level (≥1.70 mmol/L); High FG level (≥6.10 mmol/L); AH (SBP ≥140 and/or DBP ≥90 mm Hg and/or take medication for high blood pressure for at least 2 weeks).

All-cause mortality risk was significantly lower for men and women having higher HDL cholesterol level, after multivariate adjustment for age, cohort baseline survey year and other lipids (Model 1) (Table 3). On the contrary, the men having AH, being current or former smokers, or being classed as physically inactive during the leisure time had significantly increased risk of all-cause mortality. For women, having elevated triglycerides level, high fasting glucose level, having AH, being obese or being physically inactive during leisure time was significantly associated with an increased all-cause mortality risk. Additional adjustment for education, BMI, BP, fasting glucose level, smoking and physical activity habits (Model 2) revealed the similar results (with exception for high HDL cholesterol in men group and for elevated triglycerides level, BMI and leisure physical activity habits in women group).

**Table 3:** Adjusted all-cause mortality risk in relation to the presence of the dyslipidaemias and the other cardiovascular risk factors in population aged 45–64 (Kaunas urban population linked mortality file (1983–2008)).

Cardiovascular risk factors	MEN		WOMEN	
	Model 1	Model 2	Model 1	Model 2
	HR (95% CI)	HR (95% CI)	HR (95% CI)	HR (95% CI)
**Total cholesterol**				
<6.20 mmol/L	1 (Reference)	1 (Reference)	1 (Reference)	1 (Reference)
≥6.20 mmol/L	0.96 (0.83–1.11)	0.94 (0.79–1.11)	0.99 (0.82–1.19)	1.00 (0.79–1.28)
**Triglycerides**				
<1.70 mmol/L	1 (Reference)	1 (Reference)	1 (Reference)	1 (Reference)
≥1.70 mmol/L	1.03 (0.88–1.21)	1.11 (0.91–1.34)	**1.37 (1.12**–**1.68)**	1.08 (0.83–1.40)
**HDL cholesterol**				
<1.03 mmol/L	1 (Reference)	1 (Reference)	1 (Reference)	1 (Reference)
≥1.03 mmol/L	**0.81 (0.69**–**0.95)**	0.89 (0.74–1.07)	**0.68 (0.53**–**0.87)**	**0.69 (0.52**–**0.92)**
**Fasting glucose**				
<6.10 mmol/L	1 (Reference)	1 (Reference)	1 (Reference)	1 (Reference)
≥6.10 mmol/L	0.91 (0.73–1.13)	0.94 (0.75–1.67)	**2.08 (1.55**–**2.78)**	**1.95 (1.45**–**2.63)**
**Arterial hypertension**				
<140 and/or <90 mm Hg	1 (Reference)	1 (Reference)	1 (Reference)	1 (Reference)
≥140 and/or ≥90 mm Hg	**1.30 (1.13**–**1.51)**	**1.37 (1.15**–**1.63)**	**1.52 (1.24**–**1.87)**	**1.38 (1.07**–**1.78)**
**Smoking status**				
Non smokers	1 (Reference)	1 (Reference)	1 (Reference)	1 (Reference)
Smokers	**1.65 (1.42**–**1.92)**	**1.54 (1.29**–**1.84)**	1.28 (0.92–1.78)	1.11 (0.72–1.74)
**BMI, kg/m^2^**				
<25.0	1 (Reference)	1 (Reference)	1 (Reference)	1 (Reference)
25.0–29.9	**0.75 (0.63**–**0.89)**	**0.72 (0.59**–**0.87)**	1.17 (0.85–1.61)	1.02 (0.69–1.50)
≥30.0	0.89 (0.74–1.09)	0.83 (0.66–1.04)	**1.53 (1.13**–**2.06)**	1.20 (0.83–1.73)
**Level of education**				
Primary	1 (Reference)	1 (Reference)	1 (Reference)	1 (Reference)
Unfinished secondary	0.99 (0.81–1.22)	1.00 (0.78–1.28)	1.02 (0.79–1.31)	0.93 (0.67–1.28)
Secondary or college	**0.79 (0.64**–**0.97)**	0.82 (0.64–1.04)	0.95 (0.75–1.22)	0.79 (0.57–1.07)
University	**0.60 (0.48**–**0.75)**	**0.64 (0.49**–**0.83)**	0.74 (0.54–1.01)	**0.60 (0.40**–**0.90)**
**Leisure physical activity**				
Active	1 (Reference)	1 (Reference)	1 (Reference)	1 (Reference)
Inactive	**1.46 (1.26**–**1.69)**	**1.45 (1.22**–**1.72)**	**1.22 (1.02**–**1.47)**	1.17 (0.93–1.48)

Model 1 - HR adjusted by age, study survey year and other lipids.

Model 2 - HR adjusted by age, study survey year, education, BMI, blood pressure, fasting glucose level, smoking and physical activity habits and other lipids.

Abbreviations: CI - confidence interval; BMI – body mass index; HR - Hazard ratio; HDL - High density lipoprotein.

A similar pattern was observed for CVD mortality risk in 45–64 years persons (without previous CVD at the entry) (Table 4). After multivariate adjustment for age, cohort baseline survey year and other lipids (Model 1), the men and women having higher HDL cholesterol level (≥1.03 mml/L) had a significantly lower CVD mortality risk. However, after additional adjustment (Model 2) only women having higher HDL cholesterol level had significantly lower CVD mortality risk. Men having AH, reporting smoking or being physically inactive during leisure time and women having high fasting glucose level and AH (Model 1 and Model 2) had a significantly increased CVD mortality risk.

**Table 4:** Adjusted cardiovascular disease (CVD) mortality risk in population aged 45–64 (without previous CVD at the entry) in relation to the presence of the dyslipidaemias and the other cardiovascular risk factors (Kaunas urban population linked mortality file (1983–2008)).

Cardiovascular risk factors	MEN		WOMEN	
	Model 1	Model 2	Model 1	Model 2
	HR (95% CI)	HR (95% CI)	HR (95% CI)	HR (95% CI)
**Total cholesterol**				
<6.20 mmol/L	1 (Reference)	1 (Reference)	1 (Reference)	1 (Reference)
≥6.20 mmol/L	1.12 (0.88–1.42)	0.99 (0.74–1.31)	1.11 (0.81–1.53)	1.01 (0.66–1.54)
**Triglycerides**				
<1.70 mmol/L	1 (Reference)	1 (Reference)	1 (Reference)	1 (Reference)
≥1.70 mmol/L	1.10 (0.84–1.43)	1.05 (0.75–1.47)	1.36 (0.96–1.92)	0.86 (0.53–1.38)
**HDL cholesterol**				
<1.03 mmol/L	1 (Reference)	1 (Reference)	1 (Reference)	1 (Reference)
≥1.03 mmol/L	**0.72 (0.55**–**0.93)**	0.88 (0.65–1.19)	**0.59 (0.39**–**0.89)**	**0.55 (0.34**–**0.90)**
**Fasting glucose**				
<6.10 mmol/L	1 (Reference)	1 (Reference)	1 (Reference)	1 (Reference)
≥6.10 mmol/L	1.06 (0.74–1.52)	1.08 (0.75–1.56)	**2.59 (1.58**–**4.25)**	**2.63 (1.55**–**4.44)**
**Arterial hypertension**				
<140 and/or <90 mm Hg	1 (Reference)	1 (Reference)	1 (Reference)	1 (Reference)
≥140 and/or ≥90 mm Hg	**1.67 (1.30**–**2.15)**	**1.60 (1.19**–**2.16)**	**1.92 (1.35**–**2.74)**	**2.20 (1.36**–**3.55)**
**Smoking status**				
Non smokers	1 (Reference)	1 (Reference)	1 (Reference)	1 (Reference)
Current smokers	**1.63 (1.27**–**2.09)**	**1.73 (1.27**–**2.36)**	1.27 (0.73–2.20)	1.15 (0.55–2.41)
**BMI, kg/m^2^**				
<25.0	1 (Reference)	1 (Reference)	1 (Reference)	1 (Reference)
25.0–29.9	0.80 (0.59–1.09)	0.84 (0.58–1.20)	1.04 (0.62–1.72)	0.83 (0.44–1.59)
≥30.0	**1.39 (1.01**–**1.92)**	1.32 (0.89–1.96)	1.31 (0.81–2.11)	0.88 (0.48–1.62)
**Level of education**				
Primary	1 (Reference)	1 (Reference)	1 (Reference)	1 (Reference)
Unfinished secondary	1.17 (0.83–1.65)	0.94 (0.63–1.41)	1.38 (0.90–2.10)	1.62 (0.93–2.82)
Secondary or college	0.81 (0.57–1.14)	0.80 (0.54–1.19)	1.14 (0.75–1.73)	1.11 (0.64–1.97)
University	**0.63 (0.44**–**0.92)**	0.65 (0.42–1.01)	0.68 (0.39–1.20)	0.62 (0.30–1.31)
**Leisure physical activity**				
Active	1 (Reference)	1 (Reference)	1 (Reference)	1 (Reference)
Inactive	**1.36 (1.07**–**1.73)**	**1.51 (1.12**–**2.04)**	1.33 (0.98–1.82)	1.03 (0.68–1.52)

Model 1 - HR adjusted by age, study survey year, and other lipids.

Model 2 - HR adjusted by age, study survey year, education, BMI, blood pressure, fasting glucose level, smoking and physical activity habits and other lipids.

Abbreviations: CI - confidence interval; BMI – body mass index; HR - Hazard ratio; HDL - High density lipoprotein.

Adjusted by age, study survey year, education, BMI, BP, fasting glucose level, smoking and physical activity habits and other lipids all-cause and CVD mortality risks in 45–64 years persons with dyslipidaemias and a combination of other cardiovascular risk factors are shown in Table 5 and in Table 6. In men and women mixed dyslipidaemias (reduced HDL cholesterol level (<1.03 mmol/L) plus elevated triglycerides level (≥1.70 mmol/L)) significantly increased all-cause (Table 5) and CVD mortality risk (Table 6). What is more, these dyslipidaemia combinations together with the other risk factors such as AH or high fasting glucose level increased men's and women's all-cause and CVD mortality risk. Dyslipidaemias plus smoking increased men's all-cause and CVD mortality risk as compared to never smokers without these dyslipidaemias, but no association was seen among women. However, dyslipidaemias plus obesity increased women's all-cause and CVD mortality risk and men's CVD mortality risk, as compared to responders without obesity and these dyslipidaemias. As expected, the highest risk for all-cause and CVD mortality were among responders who have mixed dyslipidaemias plus high fasting glucose level, compared to responders without these risk factors.

**Table 5:** Adjusted all-cause mortality risk in population aged 45–64 in relation to the presence of the dyslipidaemias and the other cardiovascular risk factors (Kaunas urban population linked mortality file (1983–2008)).

Dyslipidaemias and other CVD risk factors combinations	MEN	WOMEN
	HR (95% CI)	HR (95% CI)
**HDL cholesterol (HDL chol) and triglycerides (TG)**		
High HDL chol + normal TG	1 (Reference)	1 (Reference)
Reduced HDL chol or elevated TG	1.06 (0.89–1.27)	1.02 (0.79–1.32)
Reduced HDL chol + elevated TG	**1.30 (1.00**–**1.68)**	**1.83 (1.26**–**2.66)**
**HDL chol, TG, and hypertension (AH)**		
No AH + high HDL chol + normal TG	1 (Reference)	1 (Reference)
No AH + (reduced HDL chol or elevated TG)	0.95 (0.72–1.26)	1.39 (0.90–2.13)
AH + (reduced HDL chol or elevated TG)	**1.30 (1.05**–**1.61)**	**1.49 (1.11**–**2.01)**
AH + reduced HDL chol + elevated TG	**1.71 (1.23**–**2.38)**	**2.56 (1.61**–**4.05)**
**HDL chol, TG and fasting glucose (FG)**		
Normal FG + high HDL chol + normal TG	1 (Reference)	1 (Reference)
Normal FG + (reduced HDL chol or elevated TG)	1.08 (0.90–1.30)	1.11 (0.86–1.45)
High FG + (reduced HDL chol or elevated TG)	0.87 (0.68–1.13)	**1.71 (1.20**–**2.44)**
High FG + reduced HDL chol + elevated TG	**1.76 (1.14**–**2.71)**	**4.43 (2.72**–**7.20)**
**HDL chol, TG and smoking status**		
Never smokers + high HDL chol + normal TG	1 (Reference)	1 (Reference)
Never smokers + (reduced HDL chol or elevated TG)	1.03 (0.77–1.38)	1.07 (0.83–1.38)
Smoking + (reduced HDL chol or elevated TG)	**1.57 (1.26**–**1.96)**	1.21 (0.75–1.94)
Smoking + (reduced HDL chol + elevated TG)	**1.89 (1.34**–**2.66)**	1.25 (0.30–5.17)
**HDL chol, TG, and obesity**		
No obesity + high HDL chol + normal TG	1 (Reference)	1 (Reference)
No obesity + (reduced HDL chol or elevated TG)	1.00 (0.81–1.24)	1.14 (0.76–1.71)
Obesity + (reduced HDL chol or elevated TG)	1.23 (0.95–1.60)	1.17 (0.82–1.67)
Obesity + reduced HDL chol + elevated TG	1.11 (0.76–1.61)	**2.25 (1.42**–**3.55)**

HR adjusted by age, study survey year, education, BMI, total cholesterol level, triglycerides level, HDL cholesterol level, blood pressure, AH, FG level, smoking and physical activity habits.

Abbreviations: CI - confidence interval; BMI – body mass index; HR - Hazard ratio; SBP – systolic blood pressure; DBP - diastolic blood pressure; FG - fasting glucose; TG – triglycerides; High HDL chol - High density lipoprotein cholesterol ≥1.03 mmol/L; Reduced HDL chol - High density lipoprotein cholesterol <1.03 mmol/L; Normal TG – triglycerides <1.70 mmol/L; Elevated TG - triglycerides ≥1.70 mmol/L; Normal FG - fasting glucose level <6.10 mmol/L; High FG - fasting glucose level ≥6.10 mmol/L; No AH – no arterial hypertension (SBP <140 and/or DBP <90 mm Hg); AH - arterial hypertension (SBP ≥140 and/or DBP ≥90 mm Hg and/or take medication for high blood pressure for at least 2 weeks); No obesity – BMI <30.0 kg/m^2^; Obesity - BMI ≥30.0 kg/m^2^.

**Table 6:** Adjusted cardiovascular disease (CVD) mortality risk in population aged 45–64 in relation to the presence of the dyslipidaemias and other cardiovascular risk factors (Kaunas urban population linked mortality file (1983–2008)).

Dyslipidaemias and other CVD risk factors combinations	MEN	WOMEN
	HR (95% CI)	HR (95% CI)
**HDL cholesterol (HDL chol) and triglycerides (TG)**		
High HDL chol + normal TG	1 (Reference)	1 (Reference)
Reduced HDL chol or elevated TG	1.24 (0.80–1.93)	0.82 (0.51–1.32)
Reduced HDL chol + elevated TG	**1.15 (1.10**–**1.20)**	**2.13 (1.12**–**4.07)**
**HDL chol, TG, and hypertension (AH)**		
No AH + high HDL chol + normal TG	1 (Reference)	1 (Reference)
No AH + (reduced HDL chol or elevated TG)	0.93 (0.55–1.55)	1.58 (0.69–3.64)
AH + (reduced HDL chol or elevated TG)	**1.64 (1.13**–**2.38)**	**2.44 (1.37**–**4.35)**
AH + reduced HDL chol + elevated TG	**2.10 (1.21**–**3.64)**	**4.56 (1.94**–**10.7)**
**HDL chol, TG and fasting glucose (FG)**		
Normal FG + high HDL chol + normal TG	1 (Reference)	1 (Reference)
Normal FG + (reduced HDL chol or elevated TG)	0.99 (0.72–1.36)	0.86 (0.52–1.40)
High FG + (reduced HDL chol or elevated TG)	0.91 (0.58–1.41)	1.78 (0.94–3.37)
High FG + reduced HDL chol + elevated TG	**2.34 (1.21**–**4.53)**	**6.64 (2.99**–**14.7)**
**HDL chol, TG and smoking status**		
Never smokers + high HDL chol + normal TG	1 (Reference)	1 (Reference)
Never smokers + (reduced HDL chol or elevated TG)	1.28 (0.78–2.12)	0.92 (0.58–1.46)
Smoking + (reduced HDL chol or elevated TG)	**1.92 (1.28**–**2.87)**	0.99 (0.42–2.35)
Smoking + (reduced HDL chol + elevated TG)	**1.92 (1.02**–**3.59)**	2.81 (0.61–12.9)
**HDL chol, TG, and obesity**		
No obesity + high HDL chol + normal TG	1 (Reference)	1 (Reference)
No obesity + (reduced HDL chol or elevated TG)	1.04 (0.71–1.53)	1.23 (0.63–2.44)
Obesity + (reduced HDL chol or elevated TG)	1.67 (0.92–3.02)	0.66 (0.33–1.30)
Obesity + reduced HDL chol + elevated TG	**1.72 (1.12**–**2.63)**	**2.39 (1.10**–**5.20)**

HR adjusted by age, study survey year, education, BMI, total cholesterol level, triglycerides level, HDL cholesterol level, blood pressure, AH, FG level, smoking and physical activity habits.

Abbreviations: CI - confidence interval; BMI – body mass index; HR - Hazard ratio; SBP – systolic blood pressure; DBP - diastolic blood pressure; FG - fasting glucose; TG – triglycerides; High HDL chol - High density lipoprotein cholesterol ≥1.03 mmol/L; Reduced HDL chol - High density lipoprotein cholesterol <1.03 mmol/L; Normal TG – triglycerides <1.70 mmol/L; Elevated TG - triglycerides ≥1.70 mmol/L; Normal FG - fasting glucose level <6.10 mmol/L; High FG - fasting glucose level ≥6.10 mmol/L; No AH – no arterial hypertension (SBP <140 and/or DBP <90 mm Hg); AH - arterial hypertension (SBP ≥140 and/or DBP ≥90 mm Hg and/or take medication for high blood pressure for at least 2 weeks); No obesity - BMI<30.0 kg/m^2^; Obesity - BMI ≥30.0 kg/m^2^.

## Discussion

Lithuanian population characterised by high morbidity and mortality from CVD and a high prevalence of hypercholesterolemia [20], [21]. In our large longitudinal study we provided reliable information on dyslipidaemias and other CVD risk factors: first, we estimated the trends of the prevalence of dyslipidaemias and other selected CVD risk factors during 25 year period (1983–2008). Findings in our longitudinal study showed very few changes in the prevalence, distribution and mean levels of lipids and other analysed cardiovascular health factors during 25 years in urban adult population. In terms of cardiovascular risk profile only a few positive changes were observed: in men the prevalence of overweight decreased, although over the same period the prevalence of obese men increased; in women the prevalence of normal total cholesterol level increased. However, more changes were unfavourable: comparing 1983–1984 and 2006–2008 random samples the mean level and proportion of high level of fasting glucose increased irrespective of gender. Also, in women over the same period the prevalence of current smokers increased from 3.4 to 15.4%. However the risk profile of BP, physical activity habits, serum triglycerides and HDL cholesterol did not change in men and women during the study period. A systematic review of data from health examination surveys and epidemiological studies (199 countries and territories, and 3.0 million participants) show that globally, mean total cholesterol changed slightly between 1980 and 2008, falling by less than 0.1 mmol/L per decade in men and women aged 25 years and older [22]. Total cholesterol fell in the high-income region consisting of Australasia, North America, and Western Europe, and in central and Eastern Europe; the regional declines were about 0.2 mmol/L per decade for both sexes, with posterior probabilities of these being true declines 0.99 or greater mmol/L [22]. Data from cross-sectional US National Health and Nutrition Examination Survey, show, that between 1988–1994 and 1999–2002, mean total cholesterol declined in adults, however the geometric mean triglyceride levels increased but mean HDL cholesterol remained unchanged [23]. The results from northern Sweden study which examined trends in serum cholesterol and BMI from 1986 to 2010 show what BMI increase continuously for both sexes, whereas serum cholesterol levels decreased during 1986–2004, remained unchanged until 2007 and then began to rise [24].

In comparison, among the National Health and Nutrition Examination Survey (NHANES) III participants in the US, from 7 analysed cardiovascular health risk factors using American Heart Association (AHA) definition only in smoking were favourable changes detected: proportion of never-smokers significantly increased from 1988–1994 to 2005–2010; however for other CVD health risk factors, such as obesity, total cholesterol level ≥5.2 mmol/l, arterial hypertension unfavourable changes were detected [25].

Despite few slight positive changes of normal total cholesterol level in women group, the prevalence of dyslipidaemias in our urban population aged 45–64 years continues to be high. The data from last health survey (HAPIEE study in 2006–2008) showed that 29.1% of men and 23.8% of women had elevated triglycerides level (≥1.70 mmol/L), 13.6% of men and only 5% of women have reduced HDL cholesterol level (<1.03 mmol/L). Thus normal total cholesterol level (<5.20 mmol/L) was detected only for 29.1% of men and 22.1% of women. From the NHANES 2003–2006 data, an estimated 53.0% of US adults have at least one lipid abnormality; 23.0% had low HDL cholesterol level, approximately 30.0% had elevated triglycerides level [12]. The Trabzon lipid study in Turkey showed prevalence's of hypercholesterolemia, low HDL cholesterol, and elevated triglycerides level were 37.5%, 21.1%, and 30.4%, respectively [26]. Over the past two decades, the prevalence of dyslipidaemias shows a tendency to increase in economically developing countries [27]. However, differences in methodology between national surveys can make international comparisons difficult.

In our large longitudinal study we analysed the impact of mixed dyslipidaemias in combination with other CVD risk factors on risk of all-cause and CVD mortality in urban population aged 45–64 years. At first we analysed the impact of single dyslipidaemias on all-cause and CVD mortality risk in men and women populations. After multivariate adjustment for age, cohort baseline survey year and other lipids (Model 1), the men and women having higher HDL cholesterol level (≥1.03 mml/L) had a significantly lower all-cause and CVD mortality risk. While, the women having elevated triglycerides level (≥1.70 mmol/L) had a significantly increased all-cause mortality risk. However this association was weakened after adjustment for the other risk factors such as education, BMI, BP, fasting glucose level, smoking and physical activity habits (Model 2). The Emerging Risk Factors Collaboration also did not find a significant independent effect of triglycerides on risk of CVD, however low HDL cholesterol level was an independent risk factor for CVD [28], [29]. Another collaborative analysis including 10,269 participants from 7 studies in Europe (DECODE Study), reported that higher triglyceride level (≥1.70 vs. <1.70 mmol/L) was associated with an increased risk of CVD (but not all-cause) mortality in women and men [30]. A systematic review and meta-analysis of 61 prospective studies show that elevated triglycerides levels were dose-dependently associated with higher risks of CVD and all-cause mortality: risks of CVD and all-cause deaths were increased by 13.0% and 12.0% per 1-mmol/L triglycerides increment (p<0.001) [31].

The absence of an independent positive association of total cholesterol with all-cause and CVD mortality in our longitudinal study, is unexplained, and invites for further research. A meta-analysis of individual data from 61 prospective studies indicated, that total cholesterol was positively associated with ischaemic heart disease mortality in middle and old age groups, however the proportional risk reduction decrease with increasing BP, since the absolute effects of cholesterol and BP are approximately additive [32]. Data from the NHANES III surveys (1988–1994 to 2005–2010) showed, that low total serum cholesterol (<5.2 mmol/l) was independently associated with a significantly higher risk of all-cause mortality (HR = 1.28 (95% CI 1.15–1.42) [32].

We then analysed the impact of mixed dyslipidaemias in combination with other CVD risk factors on all-cause and CVD mortality risk. Findings in our longitudinal study showed that in men and women aged 45–64 years mixed dyslipidaemia's (reduced HDL cholesterol level (<1.03 mmol/L) plus elevated triglycerides level (≥1.70 mmol/L)) significantly increased the risk for all-cause mortality (respectively HR = 1.30 and HR = 1.83) and CVD mortality (respectively HR = 1.15 and HR = 2.13). What is more, our results indicated that these mixed dyslipidaemia combinations together with other risk factors such as AH, high fasting glucose level increased all-cause and CVD mortality risk in men and women. These mixed dyslipidaemias plus smoking increased all-cause and CVD mortality risk only in men compared to never smokers without these dyslipidaemias. However, these dyslipidaemias plus obesity increased all-cause and CVD mortality risk in women and CVD mortality risk in men, as compared to responders without obesity and these dyslipidaemias.

Based on evidence available at the time, these two lipid abnormalities such as low HDL cholesterol concentration and elevated triglycerides concentration together with the other risk factors such as AH, high fasting glucose level and obesity were included in definitions of the metabolic syndrome [33]. In previous analysis of Kaunas data it was shown that metabolic syndrome defined by International Diabetes Federation (IDF) and new Joint Interim Societies (JIS) definitions remained the only significant determinants for all-cause mortality and CVD mortality in men (without previous CVD); however, in women the metabolic syndrome was not associated with CVD mortality risk [34].

Smoking has been clearly established as a modulator of plasma lipid levels and as an independent risk factor for CHD. Cigarette smokers have higher cholesterol levels, lower HDL cholesterol levels and elevated triglycerides level [35]–[37]. In previous studies [36], as well as in our study, the impact of smoking on the global burden of total deaths from CVD largely different between genders due to the higher prevalence of smoking among men than women.

A major cause of low HDL cholesterol is abdominal obesity, the worldwide incidence of which is increasing at an alarming rate [1]. Data from our study shows that these dyslipidaemias plus obesity increased women's all-cause and CVD mortality risk and men's CVD mortality risk, as compared to responders without obesity and these dyslipidaemias.

The Australian Diabetes, Obesity, and Lifestyle Study showed that impaired fasting glucose is an independent predictor for CVD mortality after adjustment for age, sex, and other traditional CVD risk factors [38]. Data from 14 European population-based prospective studies of 9,132 men and 8,631 women aged 25–89 years (DECODE Study) showed that low HDL cholesterol increases CVD mortality in both diabetic and non-diabetic individuals defined based on the fasting glucose criteria; however, triglycerides is a significant CVD risk predictor only in the presence of combined hyperglycaemia or diabetes [39].

Our data indicate that dyslipidaemias are very common and an important health problem among the adult population 45–64 years of Lithuania. To control dyslipidaemias, effective public health education and urgent measures are essential. These results emphasize the continued need for improving education about dyslipidaemia for both patients and healthcare providers, and developing better public health prevention strategies aimed at reducing well-known dyslipidaemia risk factors, especially for these patients, who have other CVD risk factors such as hyperglycaemia, AH, and obesity. Strategies to increase the concentration of HDL cholesterol should begin with lifestyle changes such as weight reduction, increased physical activity and smoking cessation [1].

**The strength of our study includes** the prospective character, which makes selection and information bias unlikely. Numerator-denominator bias is minimized through linkage of the survey cohorts with mortality register, rather than relying on direct contact with participants or their relatives. In addition, we have adjusted for a range of potential confounding variables, including age, gender, education, BMI, physical activity level in the analyses that are presented. Another advantage is high data comparability with other studies because the definition used for the CVD risk factors was the same as recommended by AHA or used in other cohort studies.

**It is important to be aware of several limitations of our results.** First, the present study did not examine a national sample, but rather included only a random sample of 45–64 year-olds of urban population of one city. Further study is needed to examine these associations in younger and the older sections of the population.

Second, our study involved only one assessment of the included cardiovascular risk factors. Participants could have changed their lifestyle, therefore there is a potential for exposure misclassification, which may have affected our risk estimates. Despite this, most of the analyzed levels of cardiovascular risk factors were found to be predictive for both all-cause and CVD mortality. Some other cohort studies have reported that although many variables and risk factors changed over time, the baseline survey data were predictive for mortality [40].

Third, the study design did not allow us to consider the effects of all possible genetic and environmental factors and their interactions on lipid levels. Several genes such as Apolipoprotein E (APOE), the human Class B Type I Scavenger Receptor (SCARB1), Peroxisome proliferator-activated receptor-a (PPARα) genotypes were found to be associated with lipid levels and the risk of CVD. Recent data show, that in men, the APOE4 genotype and the PPARα genotype CG were correlated with an atherogenic lipid profile while the SCARB1 genotype CT had an atheroprotective effect on lipid levels; in women, APOE2 carriers had the lowest odds of high LDL cholesterol [41]. However, assessments of genetic factors were not possible across all five surveys. Healthy nutritional habits are one an important environmental determinant of lipid levels [1]. Epidemiological studies have demonstrated some positive changes in the diet of the Lithuanian population over the last few decades [42]. Positive changes in diet have contributed to a decline in serum cholesterol levels; however, the mortality rate from CHD has remained high. Diet status evaluation in these cohorts was based on food frequency questionnaire, but only few questions were possible across all five surveys. For this reason we did not include nutrition habits in statistical analyses.

Fourth, CVD is a growing problem in worldwide and traditional CVD risk factors do not account for the entirety of risk and there are many people who have events who do not fit the traditional definition of “high risk”. New emerging risk factors, both biological and genetic, are reshaping the understanding of heart disease and the approach to risk stratification. Biomarkers such as C-reactive protein (CRP), fibrinogen, and interleukin-6 are associated with CVD and death in general populations [43]. Some studies concluded that IL-6 levels, but not CRP or fibrinogen levels, add significantly to the prediction of macrovascular events and mortality in individuals with type 2 diabetes who have baseline CVD or risk factors [43]. Data from NHANES study show that red cell distribution width (RDW), but not CRP, was associated with CHD mortality independent of traditional risk factors in a cohort with no pre-existing CVD, thus RDW may be considered a stronger biomarker for CHD death than CRP and needs further prospective evaluation in CVD risk assessment [44]. However, many earlier research studies have explored the relationship between CRP and related CVD risk factors in healthy men and women; and results show that CRP one of the most extensively studied plasma inflammatory marker, has been recognized as a strong predictor for CVD [45]–[47]. Results from MONICA/KORA Augsburg Cohort Study show, that increased circulating high-sensitivity CRP concentrations (hsCRP >3 mg/L vs. those with hsCRP <1 mg/L) are associated with an increased risk of death from CVD and CHD (respectively HRs were 2.15 (95% CI 1.39–3.34) for fatal CVD, and HRs were 1.74 (1.04–2.92) for fatal CHD) [48]. Raised plasma CRP concentrations have been shown to be associated with aging, smoking, low HDL cholesterol level, and obesity [49]–[53]. Data from 51 cross-sectional studies show that obesity is associated with elevated levels of CRP, and the association is stronger in women [49]. Also, CRP appears to have a stronger negative correlation with increasing HDL cholesterol than with other lipid parameters, especially in women [50]. The large-scale, population-based NHANES III study revealed a strong independent dose-response relationship between cigarette smoking and elevated levels of CRP [53]. Recent research shows that very highly increased high-sensitivity CRP (hsCRP >10 mg/L) are strong predictor of clinical events and also are associated with several modifiable CVD risk factors, including smoking, HDL cholesterol, and central obesity [54]. Results from MONICA/KORA Augsburg case-cohort study show, that inclusion of multiple inflammation-related biomarkers into a basic model and into a model including cardiometabolic risk factors significantly improved the prediction of coronary events, although the improvement was less pronounced for the latter endpoint [55]. However in this work were not possible to analyse the influence of inflammatory factors such as CRP, on CVD risk, because we have not data about CRP or other inflammation factors in all five surveys which are presented in this article.

There is recent evidence suggesting that chronic oral inflammatory conditions may contribute to systemic inflammation [56], thus the oral health may also be associated with all-cause mortality risk [57]. One issue of particular concern is that asymptomatic chronic oral inflammatory diseases such as endodontic infections and periodontal disease are very prevalent in Lithuania [58]. However, we have not data about endodontic infections and periodontal disease in all five surveys which are presented in this article. So the present study could not evaluate oral health conditions and inflammation biomarkers as possible confounders in the analysis for all-cause and CVD mortality risk.

## Conclusion

During 25 year period (1983–2008) the prevalence of normal total cholesterol level (<5.2 mmol/L) significantly increased only in women group; however, triglycerides and HDL cholesterol did not change in men and women groups aged 45–64 years. A significant increase in the risk for all-cause and CVD mortality in men and women was associated with mixed dyslipidaemias (reduced HDL cholesterol level (<1.03 mmol/L) plus elevated triglycerides level (≥1.70 mmol/L)). These mixed dyslipidaemia combinations together with the other risk factors such as AH, high fasting glucose level were also associated with an increase in all-cause and CVD mortality risk in men and women; while, mixed dyslipidaemias plus smoking increased all-cause and CVD mortality risk only in men compared to never smokers without these dyslipidaemias; and these dyslipidaemias plus obesity increased all-cause and CVD mortality risk in women and CVD mortality risk in men, as compared to responders without obesity and these dyslipidaemia. These results emphasise the need to develop effect public health strategies to address these CVD risk factors and reduce CVD mortality.

## References

[1] European Guidelines on cardiovascular disease prevention in clinical practice (version 2012). Atherosclerosis 22: 1e68. 10.1016/j.atherosclerosis.2012.05.007 22698795

[2] World Health Organization (2009) Global Health Risks: Mortality and Burden of Disease Attributable to Selected Major Risks. Geneva: World Health Organization Press.

[3] Health in the Baltic countries 2011 (2013). 20th edition. Health Information Centre, Institute of Hygiene; Vilnius, Available: http://sic.hi.lt

[4] European Health for All Database (HFA-DB). Available: http://www.euro.who.int/hfadb Accessed 12 May 2013

[5] Grundy SM, Cleeman JI, Merz CN, Brewer HB Jr, Clark LT, et al (2004) Implications of recent clinical trials for the National Cholesterol Education Program Adult Treatment Panel III Guidelines. Circulation 110: 227–239. 15249516 10.1161/01.CIR.0000133317.49796.0E

[6] Laforest L, Ambegaonkar BM, Souchet T, Sazonov V, Van Ganse E (2012) Mixed dyslipidemias in primary care patients in France. Vasc Health Risk Manag 8: 247–254. 22566746 10.2147/VHRM.S27668 PMC3346270

[7] Silbernagel G, Schöttker B, Appelbaum S, Scharnagl H, Kleber ME, et al (2013) High-density lipoprotein cholesterol, coronary artery disease, and cardiovascular mortality. Eur Heart J 34: 3563–3571. 24014391 10.1093/eurheartj/eht343

[8] Assmann G, Schulte H (1992) Relation of high-density lipoprotein cholesterol and triglycerides to incidence of atherosclerotic coronary artery disease (the PROCAM experience). Prospective Cardiovascular Munster study. Am J Cardiol 70: 733–737. 1519522 10.1016/0002-9149(92)90550-i

[9] Barter P, Gotto AM, LaRosa JC, Maroni J, Szarek M, et al (2007) HDL cholesterol, very low levels of LDL cholesterol, and cardiovascular events. N Engl J Med 357: 1301–1310. 17898099 10.1056/NEJMoa064278

[10] Morrison A, Hokanson JE (2009) The independent relationship between triglycerides and coronary heart disease. Vasc Health Risk Manag 5: 89–95. 19436658 PMC2672447

[11] Paolillo S, Della Ratta GL, Vitagliano A, Cirillo A, et al (2013) New perspectives in cardiovascular risk reduction: focus on HDL. Monaldi Arch Chest Dis 80: 27–30. 23923587 10.4081/monaldi.2013.88

[12] Tóth PP, Potter D, Ming EE (2012) Prevalence of lipid abnormalities in the United States: the National Health and Nutrition Examination Survey 2003–2006. J Clin Lipidol 6: 325–330. 22836069 10.1016/j.jacl.2012.05.002

[13] Wietlisbach V, Marques-Vidal P, Kuulasmaa K, Karvanen J, Paccaud F, et al (2013) The relation of body mass index and abdominal adiposity with dyslipidemia in 27 general populations of the WHO MONICA Project. Nutr Metab Cardiovasc Dis 23: 432–442. 22209742 10.1016/j.numecd.2011.09.002

[14] Cullen P, Schulte H, Assmann G (1998) Smoking, lipoproteins and coronary heart disease risk. Data from the Munster Heart Study (PROCAM). Eur Heart J 19: 1632–1641. 9857915 10.1053/euhj.1998.1086

[15] Klop B, Elte JW, Cabezas MC (2013) Dyslipidemia in obesity: mechanisms and potential targets. Nutrients 5: 1218–1240. 23584084 10.3390/nu5041218 PMC3705344

[16] West KM, Ahuja MM, Bennett PH, Czyzyk A, De Acosta OM, et al (1983) The role of circulating glucose and triglyceride concentrations and their interactions with other "risk factors" as determinants of arterial disease in nine diabetic population samples from the WHO multinational study. Diabetes Care 6: 361–369. 6617413 10.2337/diacare.6.4.361

[17] Peasey A, Bobak M, Kubinova R, Malyutina S, Pajak A, et al (2006) Determinants of cardiovascular disease and other non-communicable diseases in Central and Eastern Europe: Rationale and design of the HAPIEE study. BMC Public Health 6: 255 10.1186/1471-2458-6-255 17049075 PMC1626086

[18] Third Report of the National Cholesterol Education Program (NCEP) (2002) Expert panel on detection, evaluation and treatment of high blood cholesterol in adults (Adult Treatment Panel III) final report. Circulation 106: 3143–3342. 12485966

[19] Norkus A, Ostrauskas R, Sulcaite R, Baranauskiene E, Baliutaviciene D (2000) Classification and diagnosis of diabetes mellitus (methodology recommendations). Lith Endocrinol 3: 234–241.

[20] The World Health Organization Database (2012) Available: http://www.who.int/gho/countries/en. Accessed 21 August 2012.

[21] The World Health Organization: Multinational Monitoring of trends and determinants in Cardiovascular disease (MONICA) project monograph database. Available: http://www.ktl.fi/monica/public/monograph.html. Accessed 21 August 2012.

[22] Farzadfar F, Finucane MM, Danaei G, Pelizzari PM, Cowan MJ, et al (2011) National, regional, and global trends in serum total cholesterol since 1980: systematic analysis of health examination surveys and epidemiological studies with 321 country-years and 3•0 million participants. Lancet 377: 578–586. 21295847 10.1016/S0140-6736(10)62038-7

[23] Carroll MD, Kit BK, Lacher DA, Shero ST, Mussolino ME (2012) Trends in lipids and lipoproteins in US adults, 1988–2010. JAMA 308: 1545–1554. 23073951 10.1001/jama.2012.13260

[24] Johansson I, Nilsson LM, Stegmayr B, Boman K, Hallmans G, et al (2012) Associations among 25-year trends in diet, cholesterol and BMI from 140,000 observations in men and women in Northern Sweden. Nutr J 11: 40 10.1186/1475-2891-11-40 22686621 PMC3489616

[25] Yang Q, Cogswell ME, Flanders WD, Hong Y, Zhang Z, et al (2012) Trends in cardiovascular health metrics and associations with all-cause and CVD mortality among US adults. JAMA 307: 1273–1283. 22427615 10.1001/jama.2012.339 PMC9004324

[26] Erem C, Hacihasanoglu A, Deger O, Kocak M, Topbas M (2008) Prevalence of dyslipidemia and associated risk factors among Turkish adults: Trabzon lipid study. Endocr 34: 36–51. 10.1007/s12020-008-9100-z 19003544

[27] Fuentes R, Uusitalo T, Puska P, Tuomilehto J, Nissinen A (2003) Blood cholesterol level and prevalence of hypercholesterolaemia in developing countries: a review of population-based studies carried out from 1979 to 2002. Eur J Cardiovasc Prev Rehabil 10: 411–419. 14671463 10.1097/01.hjr.0000085247.65733.4f

[28] Di Angelantonio E, Sarwar N, Perry P, Kaptoge S, Ray KK, et al (2009) Major lipids, apolipoproteins, and risk of vascular disease. JAMA 302: 1993e2000. 19903920 10.1001/jama.2009.1619 PMC3284229

[29] Barter P (2011) HDL-C: Role as a risk modifier. Atherosclerosis (Supplements 12) 3: 267–270. 10.1016/S1567-5688(11)70885-6 22152280

[30] The DECODE Study Group (2006) Comparison of different definitions of the metabolic syndrome in relation to cardiovascular mortality in European men and women. Diabetologia 49: 2837–2846. 17021922 10.1007/s00125-006-0438-6

[31] Liu J, Zeng FF, Liu ZM, Zhang CX, Ling WH, et al (2013) Effects of blood triglycerides on cardiovascular and all-cause mortality: a systematic review and meta-analysis of 61 prospective studies. Lipids Health Dis 12: 159. 24164719 10.1186/1476-511X-12-159 PMC4231478

[32] Prospective Studies Collaboration, Lewington S, Whitlock G, Clarke R, Sherliker P, et al (2007) Blood cholesterol and vascular mortality by age, sex, and blood pressure: a meta-analysis of individual data from 61 prospective studies with 55,000 vascular deaths. Lancet 370: 1829–1839. 18061058 10.1016/S0140-6736(07)61778-4

[33] Third Report of the National Cholesterol Education Program (NCEP) expert panel on detection, evaluation and treatment of high blood cholesterol in adults (Adult Treatment Panel III) final report (2002). Circulation 106: , 3143–3421. 12485966

[34] Luksiene D, Baceviciene M, Jureniene K, Bernotiene G, Reklaitiene R, et al (2012) All-cause and cardiovascular mortality risk estimation using different definitions of metabolic syndrome in Lithuanian urban population. Prev Med 55: 299–304. 23046635 10.1016/j.ypmed.2012.08.002

[35] Garrison RJ, Kannel WB, Feinleib M, Castelli WP, McNamara PM, et al (1978) Cigarette smoking and HDL cholesterol: the Framingham offspring study. Atherosclerosis 30: 17–25. 209795 10.1016/0021-9150(78)90149-1

[36] Nakamura K, Nakagawa H, Sakurai M, Murakami Y, Irie F, et al (2012) Influence of smoking combined with another risk factor on the risk of mortality from coronary heart disease and stroke: pooled analysis of 10 Japanese cohort studies. Cerebrovasc Dis 33: 480–491. 22517421 10.1159/000336764

[37] Rao Ch S, Subash Y E (2013) The effect of chronic tobacco smoking and chewing on the lipid profile. J Clin Diagn Res 7: 31–34. 23449989 10.7860/JCDR/2012/5086.2663 PMC3576744

[38] Barr EL, Zimmet PZ, Welborn TA, Jolley D, Magliano DJ, et al (2007) Risk of cardiovascular and all-cause mortality in individuals with diabetes mellitus, impaired fasting glucose, and impaired glucose tolerance: the Australian Diabetes, Obesity, and Lifestyle Study (AusDiab). Circulation 116: 151–157. 17576864 10.1161/CIRCULATIONAHA.106.685628

[39] Zhang L, Qiao Q, Tuomilehto J, Hammar N, Ruotolo G, et al (2009) The impact of dyslipidaemia on cardiovascular mortality in individuals without a prior history of diabetes in the DECODE Study. Atherosclerosis 206: 298–302. 19303072 10.1016/j.atherosclerosis.2008.12.043

[40] Clarke R, Emberson J, Fletcher A, Breeze E, Marmot M, et al (2009) Life expectancy in relation to cardiovascular risk factors: 38 year follow-up of 19,000 men in the Whitehall study. BMJ 339: b3513. 19762417 10.1136/bmj.b3513 PMC2746269

[41] Smalinskiene A, Petkeviciene J, Luksiene D, Jureniene K, Klumbiene J, et al (2013) Association between APOE, SCARB1, PPARα polymorphisms and serum lipids in a population of Lithuanian adults. Lipids Health Dis 12: 120 10.1186/1476-511X-12-120 23919842 PMC3751123

[42] Kriaucionienė V, Klumbiene J, Petkeviciene J, Sakyte E (2012) Time trends in social differences in nutrition habits of a Lithuanian population: 1994–2010. BMC Public Health 12: 218. 22436087 10.1186/1471-2458-12-218 PMC3323430

[43] Lowe G, Woodward M, Hillis G, Rumley A, Li Q, et al (2014) Circulating inflammatory markers and the risk of vascular complications and mortality in people with type 2 diabetes and cardiovascular disease or risk factors: the ADVANCE study. Diabetes 63: 1115–1123. 24222348 10.2337/db12-1625

[44] Veeranna V, Zalawadiya SK, Panaich S, Patel KV, Afonso L (2013) Comparative analysis of red cell distribution width and high sensitivity C-reactive protein for coronary heart disease mortality prediction in multi-ethnic population: findings from the 1999–2004 NHANES. Int J Cardiol 168: 5156–5161. 24016543 10.1016/j.ijcard.2013.07.109

[45] Rohde LE, Hennekens CH, Ridker PM (1999) Survey of Creactive protein and cardiovascular risk factors in apparently health men. Am J Cardiol 84: 1018–1022. 10569656 10.1016/s0002-9149(99)00491-9

[46] Barinas-Mitchell E, Cushman M, Meilahn EN, Tracy RP, Kuller LH (2001) Serum levels of C-reactive protein are associated with obesity, weight gain, and hormone replacement therapy in healthy postmenopausal women. Am J Epidemiol 153: 1094–1101. 11390329 10.1093/aje/153.11.1094

[47] Pearson TA, Mensah GA, Alexander RW, Anderson JL, Cannon RO 3rd, et al (2003) Markers of inflammation and cardiovascular disease: application to clinical and public health practice: a statement for health care professionals from the Center for Disease Control and Prevention and the American Heart Association. Circulation 107: 499–511. 12551878 10.1161/01.cir.0000052939.59093.45

[48] Koenig W, Khuseyinova N, Baumert J, Meisinger C (2008) Prospective study of high-sensitivity C-reactive protein as a determinant of mortality: results from the MONICA/KORA Augsburg Cohort Study, 1984–1998. Clin Chem 54: 335–342. 18156284 10.1373/clinchem.2007.100271

[49] Choi J, Joseph L, Pilote L (2013) Obesity and C-reactive protein in various populations: a systematic review and meta-analysis. Obes Rev 14: 232–244. 23171381 10.1111/obr.12003

[50] Tsai HJ, Tsai ACH (2008) The association of plasma C-reactive protein levels with anthropometric and lipid parameters in elderly Taiwanese Asia. Pac J Clin Nutr 17: 651–656. 19114404

[51] Kawamoto R, Kusunoki T, Abe M, Kohara K, Miki T (2013) An association between body mass index and high-sensitivity C-reactive protein concentrations is influenced by age in community-dwelling persons. Ann Clin Biochem 50: 457–464. 23880621 10.1177/0004563212473445

[52] Yanbaeva DG, Dentener MA, Creutzberg EC, Wesseling G, Wouters EF (2007) Systemic effects of smoking. Chest 131: 1557–1566. 17494805 10.1378/chest.06-2179

[53] Bazzano LA1, He J, Muntner P, Vupputuri S, Whelton PK (2003) Relationship between cigarette smoking and novel risk factors for cardiovascular disease in the United States. Ann Intern Med 138: 891–897. 12779299 10.7326/0003-4819-138-11-200306030-00010

[54] Hamer M, Chida Y, Stamatakis E (2010) Association of very highly elevated C-reactive protein concentration with cardiovascular events and all-cause mortality. Clin Chem 56: 132–135. 19884487 10.1373/clinchem.2009.130740

[55] Herder C, Baumert J, Zierer A, Roden M, Meisinger C, et al (2011) Immunological and cardiometabolic risk factors in the prediction of type 2 diabetes and coronary events: MONICA/KORA Augsburg case-cohort study. PLoS One 6: e19852 10.1371/journal.pone.0019852 21674000 PMC3108947

[56] Gomes MS, Blattner TC, Sant'Ana Filho M, Grecca FS, Hugo FN, et al (2013) Can apical periodontitis modify systemic levels of inflammatory markers? A systematic review and meta-analysis. J Endod 39: 1205–1217. 24041380 10.1016/j.joen.2013.06.014

[57] Padilha DM, Hilgert JB, Hugo FN, Bós AJ, Ferrucci L (2008) Number of teeth and mortality risk in the Baltimore Longitudinal Study of Aging. J Gerontol A Biol Sci Med Sci 63: 739–744. 18693229 10.1093/gerona/63.7.739 PMC4984838

[58] Sidaravicius B, Aleksejuniene J, Eriksen HM (1999) Endodontic treatment and prevalence of apical periodontitis in an adult population of Vilnius, Lithuania. Endod Dent Traumatol 15: 210–215. 10825828 10.1111/j.1600-9657.1999.tb00776.x

